# Shoulder proprioception following reverse total shoulder arthroplasty

**DOI:** 10.1007/s00264-020-04756-x

**Published:** 2020-08-15

**Authors:** Joanna Walecka, Przemysław Lubiatowski, Paolo Consigliere, Ehud Atoun, Ofer Levy

**Affiliations:** 1 Sport Trauma and Biomechancis Unit, Department of Traumatology, Orthopaedics and Hand Surgery, University of Medical Sciences in Poznan, ul Gorecka 30, 60-201 Poznan, Polska; 2 Rehasport Clinic, ul. Górecka 30, 60-201 Poznań, Poland; 3 Reading Shoulder Unit, Royal Berkshire Hospital and Berkshire Independent Hospital, Reading, Berkshire, UK

**Keywords:** Reverse shoulder arthroplasty, Proprioception, Joint position sense, Neuromuscular control, Cuff arthropathy

## Abstract

**Abstract:**

Joint replacement affects the proprioception, as shown in knees, elbows, and shoulder studies.

**Aim:**

The aim was to evaluate shoulder joint position sense (JPS) following reverse total shoulder arthroplasty (rTSA) for patients with cuff arthropathy.

**Methods:**

Twenty-nine patients that underwent unilateral rTSA (19 females, 10 males) and 31 healthy volunteers evaluated for JPS of shoulder using a dedicated high accuracy electronic goniometer. Error of active reproduction of joint position (EARJP) was assessed at the following reference positions: 30°, 60°, 90°, and 120° for forward flexion and abduction and 15°, 30°, and 45° for internal and external rotation in rTSA, contralateral non-operated, and control shoulders.

**Results:**

Results of EPRJP for rTSA, contralateral, and control (respectively) are as follows:Forward flexion: 30° = (8.0 ± 5.7, 9.8 ± 6.1, and 4.9 ± 3.0), 60° = (5.0 ± 2.8, 5.9 ± 2.7, and 5.1 ± 3.2), 90° = (3.1 ± 1.6, 5.5 ± 2.6, and 3.2 ± 1.4), and 120° = (3.4 ± 2.1, 5.6 ± 4.0, and 3.5 ± 1.7)Abduction: 30° = (5.2 ± 2.5, 9.1 ± 6.1, and 4.6 ± 2.3), 60° = (5.2 ± 3.6, 6.6 ± 4.1, and 5.3 ± 3.1), 90° = (3.8 ± 2.0; 7.4 ± 5.5, and 4.1 ± 1.9), and 120° = (5.3 ± 2.9, 7.7 ± 5.3, and 4.2 ± 1.9)Internal rotation: 15° = (4.3 ± 3.1, 6.2 ± 4.4, and 2.8 ± 1.2), 30° = (3.2 ± 1.9, 4.5 ± 2.3, and 3.3 ± 1.4), and 45° = (3.5 ± 2.0, 4.1 ± 1.8, and 2.8 ± 1.0)External rotation: 15° = (3.0 ± 1.7, 4.2 ± 2.2, and 3.6 ± 1.4) and 30° = (3.1 ± 1.5, 3.8 ± 2.6, and 3.4 ± 1.6)The results showed significantly better JPS (lower EPRJP) in shoulders following rTSA and normal control shoulders comparing with the patient’s contralateral shoulder. The explanation can be that rTSA improves joint kinematics and stability, which allows better muscular performance and proprioception feedback.

**Conclusion:**

Shoulders following rTSA show JPS superior to non-operated contralateral shoulders and comparable with healthy population shoulders. It seems that rTSA restores shoulder proprioception.

## Introduction

Proprioception is the sense of the relative position of one’s own parts of the body and movement [1]. Relating to the shoulder, proprioception is an important element of the physiology. It supports the stability and the coordination of the shoulder movements. Joint position sense (JPS) is one of the elements of proprioception defining the ability to sense and feel the position and movement of the joint in space [2–6].

Trauma, joint degenerative disease, chronic joint diseases, or surgery may affect proprioception [7–10]. Shoulder pain is the most common symptom of rotator cuff (RC) tears [11]. Massive rotator cuff tear gradually leads to loss of dynamic and passive shoulder stability and superior humeral head migration [12]. Arthropathy is the final stage of the shoulder destruction, which consists of progressive degenerative changes of the tendons and the entire joint [11]. In addition, deltoid muscle insufficiency is noted [13]. Patients reports pain round shoulder gird, weakness and limited active range of movement which affect their everyday living. The symptomatic rotator cuff-deficient, arthritic glenohumeral joint treatment is a challenge. Reverse total shoulder arthroplasty (rTSA) improves shoulder function in patients affected by rotator cuff arthropathy [14]. It should be noted that its purpose is to change and stabilize the center of rotation of the shoulder joint. rTSA construction also prevents upper migration of the humerus head and allows for deltoid muscle length recovery and improves its function [15]. However, shoulder arthroplasty is an invasive surgical procedure, involves an extensive capsulectomy, releases of the muscular attachments, and may disrupt shoulder neuromuscular control balance [16–18]. There are only few reports in the literature relating to proprioception and JPS after shoulder arthroplasty [10, 17–19]. The evidence is scarce and data often conflicting.

The null hypothesis of this study was that although shoulder function improves after rTSA, JPS would be inferior in the replaced shoulder when compared with the non-operated contralateral shoulder and the healthy control group.

### The aim

The aim of the study was to assess the shoulder proprioception after rTSA in patients affected by rotator cuff arthropathy. The secondary aim of the study was to correlate JPS with clinical functional scores and biomechanical parameters.

## Material and methods

Twenty-nine patients that underwent unilateral reverse total shoulder arthroplasty (rTSA group) between 2006 and 2013 were included in the retrospective study. Nineteen were females and ten males with an average age of 74 (61–85) years. All the patients were operated upon by a single surgeon. All had a minimum post-operative follow-up of two years. The indication for surgery was rotator cuff arthropathy in all the patients. Twenty patients underwent a primary rTSA, while nine patients underwent a revision procedure from hemiarthroplasty to rTSA. All the procedures were performed through an antero-superior (Neviaser-MacKenzie) approach. The same implant was used in all the patients (Verso, Innovative Design Orthopaedics, IDO, UK). The surgical technique assumes an attempt to restore if possible rotator cuff tendons, removing the capsule. All patients had normal deltoid muscle function preoperatively. All patients had a fully functional contralateral shoulder with no history of shoulder clinic referrals, active pain, or disability. All patients had the same post-operative protocol: sling for three weeks and then home-based self-performed physiotherapy instructed by a head surgeon.

The control group consisted of 27 healthy volunteers with normal shoulder function. The group included ten females and 17 males with an average group age of 24 years (range 21–29).

The study was approved by the Ethical Committee of the University of Medical Sciences in Poznan (62/15 from 08.01.2015; 796/16 from 16.06.2016). All participants were informed about the purpose of the study and the study procedure. Informed consent was obtained from all the participants included in the study.

### Study protocol

Clinical assessment was performed pre- and post-operatively in the rTSA group, measuring the active shoulder range of motion (ROM) and clinical functional scores including the Constant score (CS), the ADLER (Active Daily Living External Rotation) score [20], and the ADLEIR (Active Daily Living External and Internal Rotation) score [21]. The Constant score parameters include the following: pain, activities, range of motion (ROM), and strength [22]. JPS measurements and isometric testing were performed in the rTSA and the control groups.

Shoulder proprioception was measured as active joint position matching (JPM). It allows the evaluation of complex reflex that includes both joint position sense in space (JPS) and ability to actively control the desired joint position.

For this purpose, an electronic goniometer (Propriometer, Ostrow Wlkp, Poland) was used, designed to measure reproduction of joint position sense (JPS) with the use of a dedicated high accuracy electronic goniometer (0.1°). The specific methodology was previously developed and published [4, 5].

**Fig. 1:**
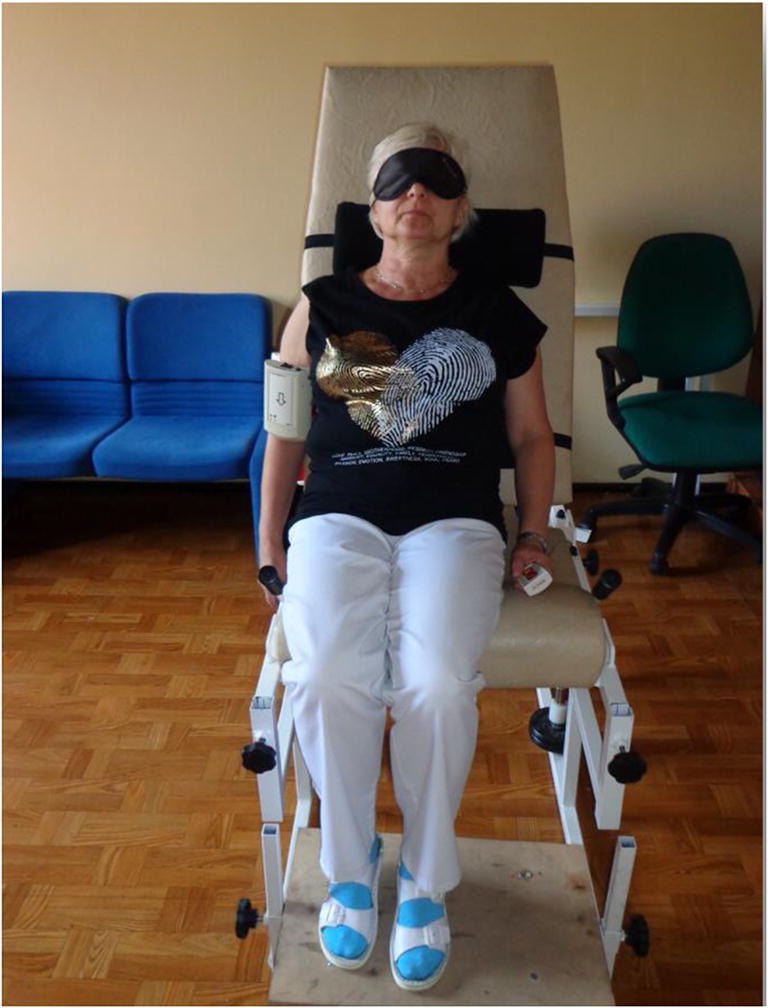
Patient position during the EARJP examination of flexion. *EARJP* error of active reproduction of joint position

The device is placed on the patient’s examined arm, and the patient is asked to actively reproduce the shoulder position in space that was previously demonstrated by the examiner (Fig. 1). The computer software allows automatic data collection. The result measured is the error of active reproduction of joint position (EARJP) [4] and calculated as absolute difference between demonstrated and reproduced angles. The average of the EARJP from 3 repetitions for each reference position was taken as the final result. JPS was measured at 14 different reference positions for each shoulder: 30°, 60°, 90°, and 120° for forward flexion and abduction and 15°, 30°, and 45° for internal (IR) and external rotation (ER) in the rTSA-operated shoulder, in the contralateral non-operated shoulder in the same group, and in both shoulders in the control group.

The isometric muscle strength was examined using an electronic dynamometer (Isometer, Innovative Design Orthopaedics, IDO, UK). Muscle strength was measured for flexion, abduction, ER, and IR in the rTSA shoulders and the control groups.

Due to the severe shoulder pain and the limitation of ROM, it was not possible to test the pre-operative proprioception and strength in the patients that underwent rTSA.

### Statistical analysis

Statistical analysis was performed by using StatPlus (AnalystSoft Inc.) for Mac OS Version 6 Excel 2011 (Microsoft). Normality tests were performed using Shapiro-Wilk test. Mann-Whitney tests and Kruskal-Wallis ANOVA were applied to compare samples. Correlation of values was assessed with the Spearman test. The level of significance was set at *p* < 0.05.

### Source of funding

The research was carried out without co-financing.

## Results

### Proprioception

Detailed results of EARJP are presented in Table 1. The statistical analysis showed significantly better joint position sense in space (JPS) (lower EARJP values) for the rTSA-operated shoulder when compared with the contralateral non-operated shoulder (in flexion 30°, 90°, and 120°; in abduction 30°, 90°, and 120°; in IR 30° and 45°; ER 15°).

**Table 1:** The results of EPRJP for rTSA, contralateral shoulders, and the healthy control group

Position		EPRJP			*p* Value (*p* < 0.05)
		rTSA (29) (O)	Contralateral (29) (NO)	Control group (27) (C)	
Forward flexion	30°	8.0 ± 5.7	9.8 ± 6.1	4.9 ± 3.0	O vs NO* O vs C** NO vs C***
	60°	5.0 ± 2.8	5.9 ± 2.7	5.1 ± 3.2	
	90°	3.1 ± 1.6	5.5 ± 2.6	3.2 ± 1.4	O vs NO*** NO vs C***
	120°	3.4 ± 2.1	5.6 ± 4.0	3.5 ± 1.7	C vs NO** NO vs C***
Abduction	30°	5.2 ± 2.5	9.1 ± 6.1	4.6 ± 2.3	C vs NO*** NO vs C***
	60°	5.2 ± 3.6	6.6 ± 4.1	5.3 ± 3.1	
	90°	3.8 ± 2.0	7.4 ± 5.5	4.1 ± 1.9	O vs NO*** NO vs C***
	120°	5.3 ± 2.9	7.7 ± 5.3	4.2 ± 1.9	O vs NO*** NO vs K***
Internal rotation	15°	4.3 ± 3.1	6.2 ± 4.4	2.8 ± 1.2	O vs NO** O vs C** NO vs C***
	30°	3.2 ± 1.9	4.5 ± 2.3	3.3 ± 1.4	O vs NO** NO vs C***
	45°	3.5 ± 2.0	4.1 ± 1.8	2.8 ± 1.0	O vs C*** NO vs C***
External rotation	15°	3.0 ± 1.7	4.2 ± 2.2	3.6 ± 1.4	O vs NO***
	30°	3.1 ± 1.5	3.8 ± 2.6	3.4 ± 1.6	

*p < 0.05, **p < 0.01, ***p < 0.001

*EARJP* error of active reproduction of joint position, *rTSA* reverse total shoulder arthroplasty

However, the ability to match the position in the rTSA-operated shoulders was inferior (higher EARJP) in 3 positions (flexion 30 °, IR 15°, and IR 45°) when compared with the control healthy group. The contralateral non-operated shoulders in the rTSA group showed inferior joint acuity when compared with the control group in most of the reference positions (flexion 30°, 90°, and 120°; abduction 30°, 90° and 120°; IR 15°, 30°, and 45°).

We observed another significant and consistent finding of a direct correlation between JPS and more elevated arm position (Tables 2, 3, and 4). We observed this tendency in each tested patient. Significant improvements were noticed for the following:

- Flexion in the rTSA shoulder (Table 2)
- Flexion and IR for the non-operated shoulder (Table 3)
- Flexion, IR, and abduction for the control group (Table 4)

**Table 2:** Comparison of the EARJP operated shoulder in the rTSA group in a certain reference position

ROM	Reference position (angle) of the operated shoulder in the rTSA group					
	30°/60°	30°/90°	60°/90°	30°/120°	60°/120	90°/120°
Flexion	*p* < 0.05	*p* < 0.05	0.05	*p* < 0.05	n.i.	n.i.
Abduction	n.i.	n.i.	n.i.	n.i.	n.i.	n.i.
	15°/30°	15°/45°	30°/45°			
IR	n.i.	n.i.	n.i.			
ER	n.i.	n.i.	n.i.			

The table shows the higher reference position, the better JPS in flexion. The *p* value of the Kruskal-Wallis ANOVA test was given in the table if it was below 0.05; *n.i.* not statistically significant, *EARJP* error of active reproduction of joint position, *rTSA* reverse total shoulder arthroplasty, *IR* internal rotation, *ER* external rotation, *ROM* range of movement

**Table 3:** Comparison of the EARJP non-operated shoulder in the rTSA group with a certain reference position

ROM	Reference position (angle) of the non-operated shoulder in the rTSA group					
	30°/60°	30°/90°	60°/90°	30°/120°	60°/120°	90°/120°
Flexion	*p* < 0.05	*p* < 0.05	*p* < 0.05	*p* < 0.05	n.i.	n.i.
Abduction	n.i.	n.i.	n.i.	n.i.	n.i.	n.i.
	15°/30°	15°/45°	30°/45°			
IR	*p* < 0.05	*p* < 0.05	n.i.			
ER	n.i.	n.i.	n.i.			

The *p* value of the Kruskal-Wallis ANOVA test was given in the table if it was below 0.05; *n.i.* not statistically significant, *EARJP* error of active reproduction of joint position, *rTSA* reverse total shoulder arthroplasty, *IR* internal rotation, *ER* external rotation, *ROM* range of movement

The table shows the higher reference position, the better JPS in flexion and internal rotation

**Table 4:** Comparison of the EARJP shoulder of the control group with a certain reference position

ROM	Reference position (angle) of the control group					
	30°/60°	30°/90°	60°/90°	30°/120°	60°/120	90°/120°
Flexion	n.i.	*p* < 0.05	*p* < 0.05	*p* < 0.05	*p* < 0.05	n.i.
Abduction	n.i.	n.i.	*p* < 0.05	n.i.	*p* < 0.05	n.i.
	15°/30°	15°/45°	30°/45°			
IR	n.i.	n.i.	*p* < 0.05			
ER	n.i.	n.i.	n.i.			

The table shows the higher reference position, the better JPS in flexion and internal rotation. The *p* value of the Kruskal-Wallis ANOVA test was given in the table if it was below 0.05; *n.i.* not statistically significant, *EARJP* error of active reproduction of joint position, *rTSA* reverse total shoulder arthroplasty, *IR* internal rotation, *ER* external rotation, *ROM* range of movement

### Clinical results and isometric strength

Significant improvements were observed post-operatively in all measured clinical parameters (pain, shoulder functional scores, and ROM). All the patients included in the study that underwent surgery were satisfied with the results, and no significant complications were observed. None of the rTSA needed to be revised within the follow-up time.

### Correlations

In the rTSA patients, some significant correlations were observed between clinical outcome and JPM. Significant negative correlation was noticed between JPM at 30° of abduction and 15° of IR and ADLER score. It means that better shoulder function (better ADLER score results) correlated with better performance on JPM (lower EARJP). A similar significant negative correlation was detected for the CS and the EARJP at 30° flexion, 45° of IR, and 30° of ER. Negative correlation was also seen between the measurements of abduction strength in 30° (Table 5). No significant correlations were noticed for ADLEIR score and pain using the Visual Analogue Scale (VAS) score (Table 5).

**Table 5:** Results of the correlation of the EARJP of shoulder operated with the Constant score results and the pain

Reference position EARJP		CONSTANT score		PAIN	
		Spearman R	*p* Value	Spearman R	*p* Value
Flexion	30°	*− 0.51*	− 0.28	− 0.28	0.17
	60°	− 0.34	− 0.13	− 0.13	0.55
	90°	0.03	− 0.03	− 0.03	0.87
	120°	− 0.09	− 0.0003	− 0.0003	0.99
Abduction	30°	− 0.28	0.28	0.28	0.17
	60°	− 0.27	− 0.06	− 0.06	0.74
	90°	− 0.02	− 0.11	− 0.11	0.6
	120°	− 0.03	0.13	0.13	0.58
Internal rotation	15°	0.02	0.23	0.23	0.29
	30°	0.18	− 0.06	− 0.06	0.8
	45°	− *0.45*	0.11	0.11	0.65
External rotation	15°	0.07	0.09	0.09	0.7
	30°	− *0.48*	0.42	0.42	0.08

*EARJP* error of active position reproduction, *Spearman R* Spearman correlation coefficient; *p* values < 0.05 are italicized

## Discussion

We found in this study that patients that underwent rTSA showed similar active joint position matching (JPM) in the operated shoulder when compared with the control healthy group (young healthy participants) and a superior JPM when compared with the contralateral non-operated shoulder in most of the tested positions.

It seems that rTSA restores the proprioceptive capability of the shoulder. These results were unexpected. Lubiatowski et al. reported the opposite findings in relation to JPM after total elbow arthroplasty [4]. It may be explained by the significant improvement of the shoulder range of motion and function and the change in the shoulder kinematics that occurs following rTSA. This improvement in shoulder function (including improved use of the deltoid muscle) restores of the shoulder proprioceptive neuromuscular control and enhances acuity to a better level than in the opposite unaffected asymptomatic shoulder.

Proprioception is a complicated issue that has been explored and investigated in various studies with occasionally contradictory conclusions [23–29]. Multiple factors have an impact on joint sense in space and motor control and are to be discussed. It is possible that a reduced neuromuscular joint movement control may lead to premature joint degeneration. This may mean that patients with inferior baseline proprioceptive control are more prone to develop osteoarthritic changes due to loss of protective mechanisms and reflexes and recurrent micro-injuries to the joint. The development of neuropathic joints in patients with syringomyelia is an extreme example [16].

On the other hand, osteoarthritis may have a negative impact on the neuromuscular control [29]. However, the information for that is limited in particular regarding the shoulder joint. There is a major obstacle to assess the proprioception of osteoarthritic shoulders pre-operatively due to the extremely painful and limited ROM.

For that reason, we could not perform pre-operative proprioception evaluation in this study, as well as in previous studies [30]. Although we expected decreased proprioceptive shoulder parameters in the study group, the correlation between proprioceptive dysfunction and degenerative joint disorder is not clear yet.

Joint position sense in osteoarthritic shoulder may be affected by multiple factors and yet not clear. Mechanoreceptors may be damaged in the joint or periarticular in degenerative joint diseases [24] or impaired by inferior joint kinematics and muscle function [16]. Joint pain [31] has been shown to disturb neurological reflexes as well. Age is another factor that needs to be considered affecting joint sense and control. Degenerative changes of the shoulder joint and rotator cuff arthropathy generally affect the elderly [29, 32, 33], as in our study (average patient’s age of 74 years).

The central nervous system is known to deteriorate with age [16, 34–36]. Goble et al. showed that JPS between 20 and 30 years of age is most accurate and gradually worsening between the ages of 35 and 50, with a marked deterioration after the age of 70 [37]. Age-related changes in the JPS may be related both to the degenerated rotator cuff tendons, condition affecting about 30% of the population over 70 years of age [38, 39], and to the aging of the nervous system.

These considerations are confirmed by our findings as the contralateral, otherwise, healthy shoulder in the rTSA group had an inferior joint acuity when compared with the younger control group (mean age 24 years).

The purpose of selecting young participants control group was to have healthy control shoulders not affected by degenerative joint or rotator cuff tear changes. Similar inclusion criteria for controls were used in previous studies [4, 26].

The reason behind the improvement observed in the joint position matching ability for the rTSA group in our study is unclear.

It is possible that the restoration of the shoulder range of movement and biomechanics improves the proprioceptive input. Proprioception inputs can originate from the joint capsule, the muscles surrounding the joint and the skin.

Some authors suggested that the joint capsule and the skin proprioceptors are less important. Anaesthetic injection into the elbow and knee joints have not shown a negative effect on joint proprioception [40, 41], surgical joint capsulectomy did not show reduced proprioception, neither.

Grigg et al. [30] investigated proprioception in 16 patients before and after total hip replacement: They did not find any difference in pre-operative and post-operative proprioception. Knee arthroplasty studies by Barrak et al. [31] and Ishii et al. [32], seem to support the observation that capsulectomy may not negatively affect proprioception, suggesting other contributors to the Joint Position Sense (JPS), such as good muscular receptors input that seems to be the most important for the proprioceptive function.

Disappearance, degeneration, or rupture of muscle fibers may cause weakness and reduced joint position sense [42]. Maier et al. [10] showed that the detachment and reconstruction of the subscapularis tendon and release of glenohumeral ligaments during shoulder arthroplasty had a detrimental effect on proprioception. Muscular damage due to eccentric exercises [33–35] or tendon degeneration may significantly impair the joint neuromuscular control. Arthroplasty surgery is invasive and involves substantial tissue dissection, a complete capsulectomy with extensive release, and significant bone resection.

In a previous study [4], on proprioception following elbow arthroplasty, the muscular damage was considered the cause of deterioration of elbow JPS. Elbow arthroplasty surgery is a traumatic procedure where not only a complete capsulectomy is performed, but also most of the muscles (forearm flexors, extensors, and triceps) are detached to obtain good exposure. However, in rTSA surgical technique, in most of the cases, an extensive tendon disruption already exists prior to surgery. The capsulectomy releases and improves substantially the range of motion, which may improve the proprioceptive input from the muscles. In our study group, an intraoperative approximation repairing the remnants of the torn cuff tendons was always attempted. Therefore, it may be that re-tensioning of the remnants of the cuff tendons and the deltoid muscle improves function [8, 36] and proprioceptive input.

We assume that the repaired remnants of the rotator cuff tendons and the improved deltoid tension (without significant collateral damage to the muscular fibres during the operation) lead to better shoulder biomechanical conditions with consequent improvement of the proprioception and neuromuscular control of the shoulder.

The few reports on proprioception in shoulder arthroplasty showed contradictory results. Cuomo at al. [8] showed improved JPS after anatomic shoulder arthroplasty, suggesting that the positive effect is related to the restoration of the shoulder anatomy and the post-operative rehabilitation protocol [8]. Kasten at al. [9], on the contrary, reported inferior joint acuity after shoulder arthroplasty; their results need to be interpreted with caution since their study group was very small (*n* = 5) and heterogeneous (both hemiarthroplasty and rTSA).

The improvement of neuromuscular control following rTSA in our study corresponds to a significant improvement in shoulder function. The rTSA showed significant improvements in all the clinical parameters (pain, ROM, clinical scores) after surgery.

We observed a positive correlation between the Constant score (CS) and the accuracy of the JPM of the operated shoulder. Maier et al. [16] investigated the relationship between pre-operative CS and proprioception in primary shoulder OA in patients who undergo anatomical shoulder arthroplasty. They showed that low pre-operative CS is a negative factor for the recovery of proprioception post-operatively.

The post-operative pain level in our study was minimal and may have contributed to better ROM and input. Unfortunately, due to significant restrictions in shoulder movement and pain, it was not possible to examine JPS before the operation in the material. What is important to underline is that Bennell et al. [38] suggested that pain is not an influencing factor for proprioception of the knee joint with diagnosed degenerative changes after examining a group of 220 patients. Similar results were shown in other studies with smaller number of patients [40, 41, 43].

Regarding function and activities of daily living (ADL), a positive correlation was found between the patient’s ADLER score and the JPM after rTSA in all tested shoulder positions.

The ADLER score evaluates the daily activities functions relying on good active external rotation such as combing hair, talking on the phone, or reaching to the mouth [37].

**Fig. 2:**
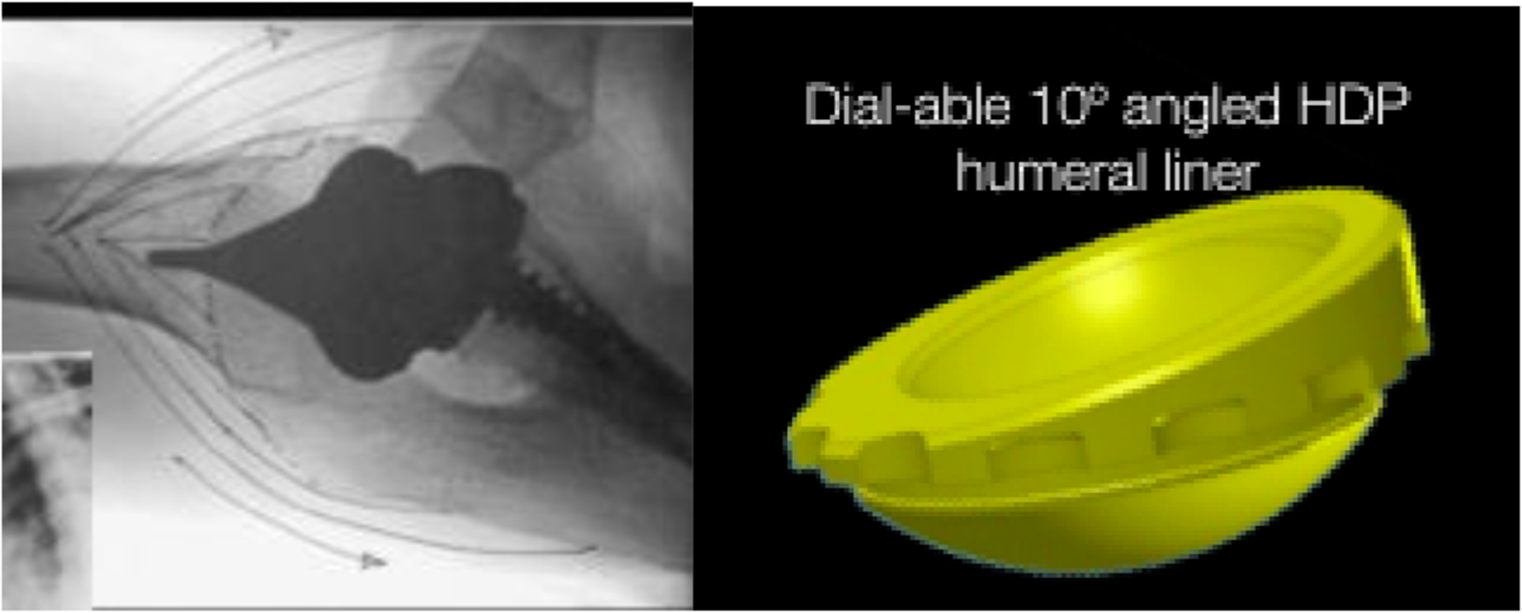
The track of the Verso implant. Polyethylene insert with a 10° angle. The engraving used with the consent of Prof. Ofer Levy

The good rotational movements observed in our study may be attributed as well to the prosthesis design. The implant used in this study uses bone cut of 55° (neck-shaft angle), but the final implant neck-shaft angle is 145° with a 10°-inclined dialable polyethylene liner (Fig. 2). The liner design reduces the possibility of impingement of the liner on the glenoid neck during rotations, therefore improving the impingement-free rotational range of motion [38] (Fig. 2).

With these findings, we may conclude that following rTSA shoulder proprioception improves and may be restored to a comparable level of normal shoulder proprioception for young control group.

The substantial improvement in shoulder movements, due to the capsulectomy and release, combined with the improved biomechanics after rTSA, improves the muscles proprioceptive mechanoreceptors input with an improvement of the JPS acuity.

### Limitations

The limitations of this study is lack of preoperative EARJP in the rTSA group. These could not be obtained in the rTSA group due to the severe pain and limitation of ROM.

The healthy controls are not age-matched, but as explained, we have considered the opposite shoulder with normal function in the rTSA group to be appropriate age-matched comparison. The young healthy volunteers control group was therefore selected to represent the normal joint without any shoulder degeneration.

## Conclusions

Shoulders following reverse TSA showed a superior joint position sense when compared with non-operated contralateral normal shoulders and were comparable with the healthy young control group. We believe that the enhanced proprioception was due to improved shoulder ROM, function, and biomechanics provided by the rTSA. There was a positive correlation of joint acuity with the clinical functional scores, ROM, and strength of the operated shoulder. There may also be age-related proprioception deterioration as shown by the lower proprioception scores observed in the non-operated shoulder in the elderly group.
